# Twelve tips on how to motivate healthcare professions students and their supervisors for Interprofessional Education

**DOI:** 10.15694/mep.2020.000243.1

**Published:** 2020-10-29

**Authors:** Cora L.F. Visser, Saskia Oosterbaan, Birgitte Mørk Kvist, Gerda Croiset, Rashmi A. Kusurkar

**Affiliations:** 1 Research in Education; 2 Gynecology & Pediatrics department OLVG West; 3 Department of Gynecology & Obstetrics; 4 Education & Training

**Keywords:** Interprofessional Education, Clinical reasoning, T-shaped professional, Self-determination theory, Scaffolding of learning, Twelve tips to make interprofessional learning motivating.

## Abstract

This article was migrated. The article was marked as recommended.

Special efforts in rotations are necessary to have students from different professions learn with, from and about each other to improve their collaboration and the quality of care. The twelve tips derived from the lived experiences and research from the authors, are intended to stimulate motivation for interprofessional education in students and their supervisors. Internalization of the value students place on interprofessional learning will improve their readiness for future interprofessional collaboration. While creating an autonomy-supportive learning environment, supervisors are capable of both scaffolding the learning of students from all professions, and learning themselves from these authentic situations. The authors promote a central place for the clinical reasoning of each profession in both the profession specific skills as well as in the communication, collaboration and team skills, thus enhancing the ‘T shaped-professional’ ( Visser, 2018).

## Introduction

Interprofessional Education (IPE) enables students from different healthcare professions to learn with, from and about each other to improve collaboration and the quality of care ( CAIPE, 2016). This learning is not an organic process, as students are frequently unsure about their roles and responsibilities and those of others they meet during rotations in the workplace. Therefore a special effort is necessary to motivate students for IPE. In healthcare education, traditionally a substantial part of the training is through workplace learning ( Dornan *et al*., 2007; Manley, Titchen and Hardy, 2009). Several reports and studies have indicated that the collaboration between healthcare professionals (HCPs) from different training and professional backgrounds is difficult ( McGrail *et al.,*2009; Lingard, 2016; Romijn *et al.,* 2018).

Interprofessional collaboration (IPC) is intended to have all HCPs share their perspectives and values in the decision-making process, both among areas of specialist expertise and with the individual patient ( Vyt, 2008). To achieve this, HCPs need get familiar with the perspectives of other healthcare disciplines and achieve interprofessional collaboration competencies. In many healthcare systems, HCPs have yet to find ways and means to enhance their IPC ( Chan & Wood, 2010; van der Lee *et al.,* 2011; Paradis & Whitehead, 2018). This article aims to give tips for IPE in the workplace, and in particular for motivating students and supervisors for IPE.

We viewed motivation for IPE from the theoretical framework of the Self-determination theory, which holds that students motivated for an activity from within, or because they find it personally important, are more likely to incorporate it into their value system ( Ryan and Deci, 2000). This internalization is essential for converting learned IPE concepts into daily practice, i.e. interprofessional collaboration (ten Cate, Kusurkar and Williams, 2011; Kusurkar *et al.*, 2013).

To enhance motivation in students, the fulfilment of the three basic psychological needs of the students is important, as described by Self-Determination Theory ( Deci and Ryan, 2002; Kusurkar and Croiset, 2015): autonomy, competence, relatedness. Typical examples of basic psychological needs fulfilment are:

• When students are in the lead, providing information about the patients and proposing the policy for their treatment, they perceive *autonomy* (choice).
• As students experience their ability to take care of patients, sharing the responsibility for them with professionals, and asking professionals outside the IPE workplace for consultations, they perceive *competence.* Bleakley and Bligh (2008) advocate a new type of apprenticeship, in which students are supported in understanding their patients themselves ( Bleakley and Bligh, 2008).
• Relatedness: When students work closely together in an IPE room on the ward, meeting students and supervisors working towards the same goals for patient care on a daily basis, mutual relations are facilitated.

By providing the feeling of choice (autonomy), of feeling able to learn how to perform the task (competence) and feeling relatedness with relevant others ( i.e. students and supervisors from all professions), an autonomy-supportive learning environment is created.

Our research and the lived experience from 2 authors (SO and BMK) form the basis for the following twelve tips. We present the tips in the order of the IPE definition: learn with, from and about (1 - 7) to improve collaboration (8 - 9) and quality of care (10 - 12).

## Tip 1: Make use of co-location to stimulate interactions

Look for opportunities to transform (part of) a ward into an IPE facility, provided that patients can be selected for the student team. Moreover, supervisors from two or more professions should be available to provide both profession specific and interprofessional supervision.

In most clinical wards, where students from different professions are co-located but learn under supervision from their own profession, IPE does not occur spontaneously. The selected patients do not have to be located in specific IPE rooms or have the same practitioner: the IPE students can ‘travel’ to the selected patients.

Traditionally, orthopaedic, obstetric or geriatric wards are chosen for IPE in Sweden and Denmark ( Jakobsen, 2016; Oosterom *et al.*, 2019). Our experience is with an internal medicine ward and an obstetric ward ( Visser *et al.*, 2019; Visser *et al.*, 2020).

## Tip 2: Create authentic learning situations to engage students

To effectively involve students in IPE, first and foremost you need to create or use an authentic learning situation. This can be done through the introduction of a real patient, either on paper (case study) or in an IPE ward. The students have to solve a problem that is perceived to be close enough to the work reality and the demands of the professions. This puts the problem in the context of an immediate application in work and hence stimulates autonomous motivation for the activity. Charles, Bainbridge and Gilbert (2010) refer to this learning environment as ‘immersion’: students are in the process of acquiring the core knowledge and skills of their profession, while developing a sense of themselves as practitioners and at the same time growing in confidence as professionals ( Charles, Bainbridge and Gilbert, 2010).

## Tip 3: Ask supervisors to scaffold the learning of students from all professions

Distinguish which professionals work on the ward frequently and are used to supervising students from their own profession. Check if these clinicians are scaffolding the learning from students of their own profession. This means that clinicians provide just-in-time and tailored guidance to students who can perform a task that they would not be able to without the supervisors’ support.

In general, scaffolding of learning is intended to a) help students remain on track; b) apply cognitive structures; c) concentrate on parts they can perform while the supervisor temporarily takes over the parts beyond their current capability; d) start with and adhere to a task; e) build on existing understanding and f) control their frustration.

In their scaffolding framework, Van de Pol *et al*. distinguish the above six intentions in support of students’ metacognitive and cognitive activities as well as student’s affect. Furthermore, the authors describe six means for supervisors to flesh out these intentions: giving feedback, providing hints to activate the groups’ thinking, instructing, explaining, modelling of key behaviours, and questioning, i.e. asking in such a way that students need to actively formulate a cognitive answer ( van de Pol, Volman and Beishuizen, 2010).

We found that clinicians are willing as well as able to scaffold the learning of students from other professions, even for the student’s profession specific clinical reasoning ( Visser *et al.*, 2020).

## Tip 4: Enhance collaboration among supervisors in the workplace

IPE workplaces offer an authentic learning situation for supervisors as well. In our studies, IPE ward supervisors experienced that observing their peers had helped them to appreciate how IPE enhanced their own skills and attitudes, clarifying their professional responsibilities and underlining the importance of interprofessional communication ( Visser *et al.*, 2019; Visser *et al.*, 2020). Supervisors elsewhere self-reported an improvement in their collaboration with supervisors from other professions ( Barr, 2002).

Paradis and Whitehead (2018) suggest that interprofessional practice offers a combination of authentic learning situations and involvement of professionals who are in a position to change the culture of healthcare in ‘a logistically more straightforward and less costly manner’ than schools and universities ( Paradis and Whitehead, 2018).

## Tip 5: Create involvement with the project

Rather than representing a specific function, the project coordinator should be someone with interprofessional skills and the ability to create an autonomy-supportive working situation for the project participants. Seek the commitment of the management for a pilot, making the initiative a project with other allowances than the usual care structure.

During the initial phase, the person or group initiating the IPE workplace should be able to perform different tasks, such as developing materials and ‘marketing the idea’, e.g. by making an information booklet for students, as well as giving presentations for managers. After some time, i.e. in a later phase, the project initiators should feel confident enough to hand over these tasks to others; sharing the responsibility with new members can enhance the sustainability of the IPE workplace ( Grymonpre *et al.*, 2016). Should the setup of the IPE workplace come under threat, all involved can help to make adaptations while sustaining its essential characteristics.

## Tip 6: Train the supervisors

Offer training sessions for intended interprofessional supervising professionals. Train them concerning learning and supervising/teaching styles and group dynamics. This can be done using the scaffolding framework (see: Tip 3), so supervisors can consciously use the six intentions and six means to put their intentions into actions.

Encourage supervisors to make suggestions regarding organisation, execution and sustainance of the IPE workplace. This can be facilitated through supervisor meetings, or during lunch or informal meetings. Offering supervisors an opportunity to act as “critical friends” stimulates a safe learning environment, where students dare to develop ( Hall and Zierler, 2015). Tip 6 and tip 4 enhance the learning of the supervisors and the fulfilment of their basic psychological needs.

## Tip 7: Stimulate positive group dynamics

Allow time and space for students to get acquainted with each other. Introducing the team phases ‘forming, storming, norming, and performing’ described in Tuckman’s model ( Bonebright, 2010) is a practical way to explain to students how group dynamics can evolve when students who are not acquainted and from different professions are combined in an interprofessional team.

Students in our IPE wards recollected the team stages vividly and were proud that within 3 weeks their team had reached the ‘performing’ stage ( Visser *et al.*, 2019; Visser *et al.*, 2020). We found that supervisors supported the students who felt they were stuck in communication because they perceived themselves as having lower status and less power. This support can be considered to guide students to reach an open exchange of relevant interpretations (Tuckman’s task activity 3, ( Tuckman and Jensen, 1977)).

## Tip 8: Focus on learning-oriented teaching

Teach students the content (what) and the reason (why), together with the metacognitive skills to monitor their interprofessional learning (how to learn), and deal with frustration when the learning gets though ( ten Cate *et al.*, 2004). Applying the principles of Learning-Oriented-Teaching model to IPE initiatives, the authenticity of learning situations clearly provides students with an unequivocal answer to the question ‘Why learn?’.

Encourage students to gradually increase self-guidance: first guidance by the teacher/supervisor, then guidance shared by student and teacher/supervisor and lastly self-guidance by the student. ( Blue *et al.*, 2010). An increasing amount of self-guidance by the student should correlate with a decreasing amount of guidance by the teacher or supervisor ( ten Cate *et al.*, 2004; Visser *et al.*, 2019). The setting of an IPE workplace stimulates students to guide their own learning by being with other students, which makes asking questions more natural ( Visser *et al.*, 2019). Activating learning by giving students assignments to collect more information on diseases or protocols, is another contribution to learning-oriented teaching ( Visser *et al.*, 2020).

## Tip 9: Stimulate ownership of learning to enhance readiness for IPE

Readiness for interprofessional learning is often used as an indicator of change in attitude towards IPE, measured before and after an IPE initiative ( Parsell and Bligh, 1999) on four dimensions: (1) roles and responsibilities of self and others, (2) knowledge and skills for teamwork, (3) benefits to patients, practice and personal growth, and (4) values. Engaging students in IPE and thus influencing their attitudes towards interprofessional collaboration (IPC) is important, because negative attitudes can stand in the way of learning. In our literature review, readiness for IPE was found to fluctuate over the years of training ( Visser *et al.*, 2017). Widening our perspective from ‘readiness for IPE’ to ‘readiness for learning’ provided us with a new clue.

Within the concept ‘readiness for learning’, Conley and French introduced ‘ownership of learning’, which encompasses motivation and engagement, goal orientation and self-direction, self-efficacy and self-confidence, metacognition and self-monitoring and persistence ( Conley and French, 2014). In our 12 tips, we already focus on the motivation and engagement of students in the IPE workplace. For their goal-orientation and self-direction it is important to let students work on a shared goal: quality of care for an individual patient. To enhance goal-sharing, students should be introduced to diverse perspectives, which are exemplified e.g. by providing relevant protocols for different professions in a booklet, providing structure for the patient care meeting reflecting the clinical reasoning process, selecting the patients with a focus on pathophysiological aspects in the beginning, followed by a focus on psychological-sociological aspects.

## Tip 10: Integrate professional learning goals and interprofessional objectives

In the IPE initiatives we studied, students felt responsible for patient-management plans. Students were expected to use their profession specific knowledge and skills and combine these with the knowledge and skills from others. Supervisors were encouraged to foster interprofessional collaboration, e.g. by directing a question from a student from one profession to a student from another profession, who might have the relevant answer.

In the literature, it appears that medical students in particular find the interprofessional learning objectives to be in competition with their profession-specific learning objectives ( Visser *et al.*, 2017). The students want opportunities to train and show their professional skills. Seen from a Self-determination theory stance, these students express their need for autonomy and competence.

In some IPE wards, students are expected to provide general care, i.e. helping patients with basic care needs. A review of Scandinavian and Danish IPE wards is inconclusive about the benefits or pitfalls of students providing general care ( Jakobsen, 2016). Looking at this aspect through the Self-determination theory lens, providing basic care in interprofessional student pairs offers opportunities for relatedness between the students and between students and patients. Thereby we would label general care as a means for IPE, not a goal in itself: We found medical students to be more confident to assess patients’ condition and nursing students felt able to explain their rationale for the care to other students ( Visser *et al.*, 2020).

## Tip 11: Demonstrate the interdependence of all professions

Sharing clinical reasoning within one’s profession is a practical way to express the perspective of that profession and can bring about a feeling of competence: ‘I’m capable of thinking in a professional way and I experience competence in the professional content’. Sharing one’s clinical reasoning with other professions make students realize what each profession adds to the patient care.

In an internal medical ward, students indicated that before the IPE initiative, they had a poor awareness of the clinical reasoning of other professions; gaining insight into others’ clinical reasoning enabled them to see ‘the pieces of the puzzle’ the patient forms for each profession and all professions involved. To be familiar with the clinical reasoning of other professions helped students to see new lines of thinking and how professionals come to certain interventions. The supervisors involved also valued the insights gained from observing the clinical reasoning of other professions during the patient care meeting ( Visser *et al.*, 2019).

This finding regarding the clinical reasoning of other professions could explain why a traditional rotation on a ward, even though students and supervisors from other professions are present, does not result in interprofessional learning. Unless the clinical reasoning of a profession is clearly articulated, others who are unfamiliar with the cognitive processes involved may not follow what is happening ( Nisbet, Lincoln and Dunn, 2013). Through the scaffolding of their clinical reasoning, the student teams can eventually devise interprofessional care plans for patients presenting problems that go beyond the capabilities of the individual students and of a single profession. Students then experience the interdependence of professions and learn about the roles and responsibilities of other professions.

## Tip 12: Consider clinical reasoning the heart of the T-shaped professional

To support the clinical reasoning of all health professions the patient care meeting in an IPE workplace should be structured. This structure should contain the following steps: assessment of the current situation, generating a problem inventory, performing a differential diagnosis, putting together a care plan, deciding on interventions, following up, and summarising. The structure clarifies how a student is expected to present a patient to the team: which basic information is needed, in a concise manner, before a student or the team can communicate ideas for the decision making and management of patient problems. The benefits of providing a structure for interprofessional meetings are well acknowledged in the literature ( Hudson, 2009; Baker *et al.*, 2011; Goldman *et al.*, 2015). Students will recognize that being able to reason about the clinical problems forms part of their professional skills. But collaboration is also dependent on good communication.

**Figure 1.**
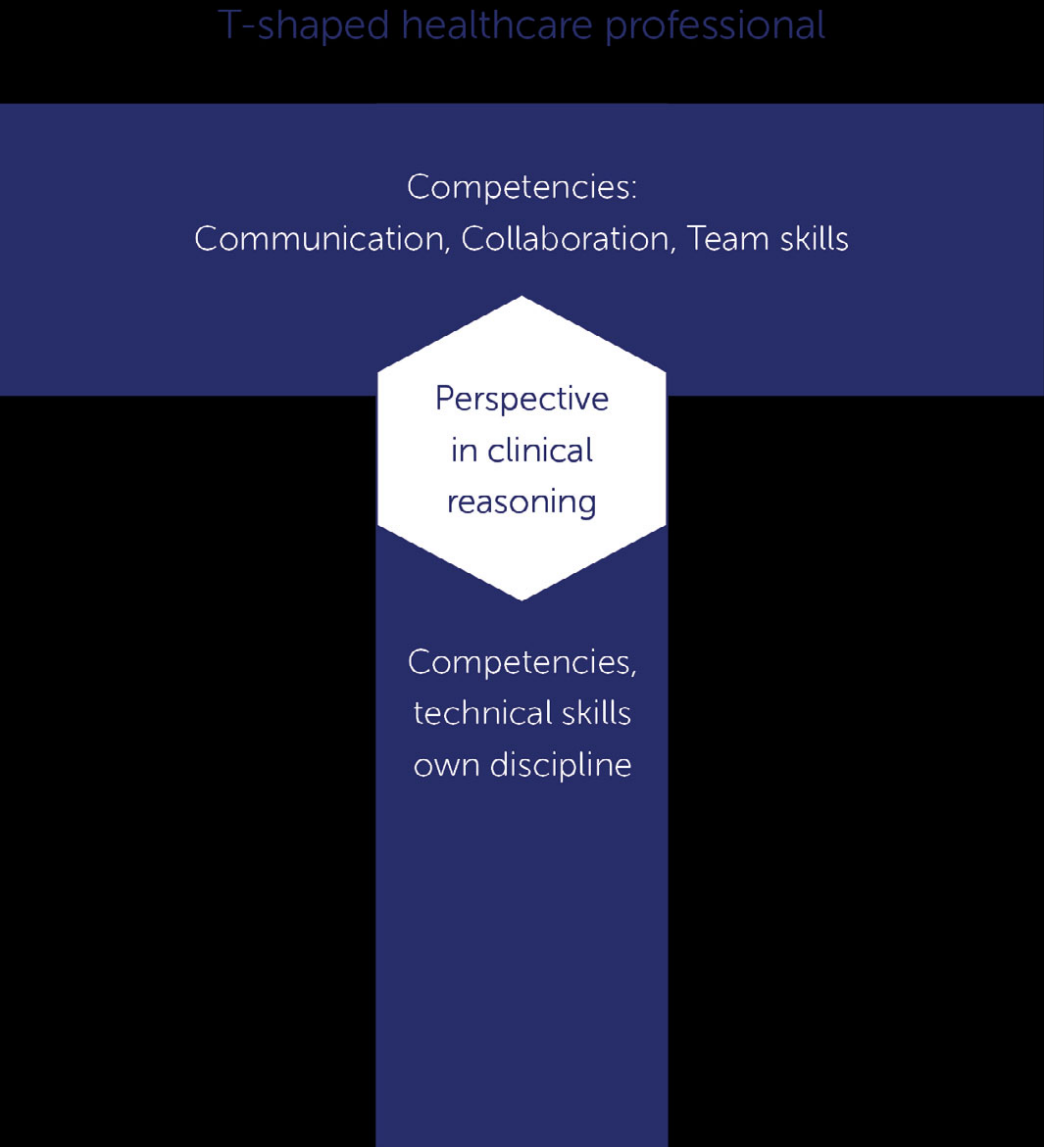
Clinical reasoning of a profession placed where the profession-specific and the general competencies meet.

The T- shaped professional is a metaphor to describe the combination of profession specific abilities in the vertical bar of the T-shape and the ability to communicate with experts in other professions in the horizontal bar. Education for medical, nursing, and allied health professions in many countries is based on the CanMEDS roles and competencies ( Frank, Snell and Sherbino, 2015). In their analytical process, different professions take different components of the patient situation into consideration ( Trede and Higgs, 2008). When professionals with different backgrounds are able to combine their perspectives, ‘communication, collaboration and complementarity lead to IPC’ ( Engel, 1977). Through thinking and communicating, clinical reasoning forms the linking pin between the profession-specific abilities and the communication skills. This professional perspective is therefore placed in the heart of the T-shape ( Figure 1). The central place for clinical reasoning implies that students participating in an IPE workplace need to master enough skills to perform their profession-specific clinical reasoning and to communicate about it ( Visser, 2018).

Students and their supervisors who can combine the depth of expertise and related skills in a single field (vertical bar of T-shape), with the ability to collaborate interprofessionally with experts in other areas (horizontal bar) can become T-shaped professionals - Figure 1 ( Donofrio, Spohrer and Zadeh, 2010).

With these twelve tips, we aimed at enhancing the intrinsic motivation of **all** stakeholders in IPE, by highlighting the vital role of clinical reasoning and of their ownership of learning.

## Conclusion

The communication and team skills of the T-shaped professional are emphasised when students share their professional specific clinical reasoning to discuss patients’ problems. Scaffolding of their learning helps them to experience the interdependence of professions to come up with patient management plans that no student or profession could have planned alone, while stimulating their autonomy, competence and relatedness. These twelve tips are expected to positively impact on the affective component of IPE and the motivation of students and their supervisors for interprofessional education, internalizing the IPE to become future IPC.

## Take Home Messages

• Explain to students how group dynamics can evolve when students who are not acquainted and from different professions are combined in an interprofessional team.
• To have students from different professions learn with each other on a ward, insight into their own and others’ clinical reasoning is necessary.
• Structure how a student is expected to present a patient to the team before ideas for the decision making and management of patient problems can be communicated.
• The project coordinator for an Interprofessional Eduation ward should be someone with interprofessional skills and the ability to create an autonomy-supportive working situation for the project participants.

## Notes On Contributors

**Cora L.F. Visser**, PhD, is trained as a dietitian and educationalist and completed her PhD thesis regarding the affective component of Interprofessional Education in 2018. She works as an educationalist for the Institute of Education and Training Amsterdam University Medical Center, location VUmc, Amsterdam, the Netherlands. ORCID ID: https://orcid.org/0000-0001-9694-6869

**Saskia Oosterbaan**, Msc, MD, is the project coordinator of an Obstetric IPE ward and a Geriatric IPE ward, OLVG West, Amsterdam, the Netherlands.

**Birgitte Mørk Kvist**, Master of Learning Processes, is a midwife and an educator of healthcare professionals regarding IPE, Department of Gynecology & Obstetrics, Regional Hospital Herning, Herning, Denmark. She is the project coordinator of an Obstetric IPE ward.

**Gerda Croiset**, PhD, MD, is dean of Education and Training, University Medical Center Groningen, Groningen, the Netherlands.

**Rashmi A. Kusurkar**, MD, PhD, FAMEE, is an expert on SDT, Associate Professor and Research Programme Leader at Research in Education, Amsterdam UMC Faculty of Medicine, Vrije Universiteit, Amsterdam, the Netherlands. ORCID ID: https://orcid.org/0000-0002-9382-0379
