# Supplementary material for: Twelve tips on how to motivate healthcare professions students and their supervisors for Interprofessional Education
Source: MedEdPublish (2016). 2020 Oct 29;9:243. [Version 1] doi: 10.15694/mep.2020.000243.1 (PMC10697523; doi:10.15694/mep.2020.000243.1)
Supplement: Peer review history

# Reviewer report: Reviewer response for version 1

**DOI:** 10.21956/mep.20183.r31214

**Published:** 2020-12-06

**Authors:** Ardi Findyartini

**Affiliations:** 1 Indonesia Medical Education and Research Institute

This review has been migrated. The reviewer awarded 4 stars out of 5 Thank you for the opportunity to review the paper. Congratulations Cora and all authors!I find the paper very interesting and has elaborated important tips in motivating students and supervisor in interprofessional education (IPE). IPE conducted in hospital setting is very valuable since it gives health professions students direct and real experience. The tips in this paper are well built on this learning opportunity. The linkages between interprofessional collaborative practice (IPC) and IPE can be strengthen in the introduction. The paper provides insights on how motivation (using SDT theory as the key conceptual framework) can be nurtured through the IPE implementation in the hospital. Regarding this, I would expect how each tip links to the SDT theory, and what are the implications.Regarding the tips, my only note is on the tip 10 - Integrate professional learning goals and interprofessional objectives. I wonder if this tip should be the first tip, since the goal definition may influence further decisions on the co-location, co-supervision, the scaffolding processes and overall IPE implementation in the teaching hospital.I appreciate the use of T-shape professionalism concept. In addition, what fascinates me and really triggers further thoughts on faculty development in IPE is the need of good co-supervision with ability to nurture collaboration competence and other clinical skills, especially clinical reasoning skills of different health professions.Overall, I find that the the paper is very insightful and has attempted to link evidence to the best practice. The tips are very useful in supporting the implementation of rich learning experience of IPE in the real healthcare setting.

Reviewer Expertise:

NA

No decision status is available

# Reviewer report: Reviewer response for version 1

**DOI:** 10.21956/mep.20183.r31213

**Published:** 2020-12-01

**Authors:** Helena Filipe

**Affiliations:** 1 Hospital of the Armed Forces/PL-EMGFA

This review has been migrated. The reviewer awarded 4 stars out of 5 Thank you for the opportunity to review this interesting paper. Authors picked up a topic on the spotlight. Combining their experience and the literature review they complied a set of twelve guidelines on Interprofessional Education in undergraduate medical education. The chosen format is very attractive for clinical educators and perfect for those wishing to improve their skills and start a structured project on Interprofessional Education (IPE), undergraduate education (UGE) level forward.They have organised the tips into in 3 groups a) to learn with, from and about (tips 1 - 7); b) to improve collaboration (tips 8 - 9); and c) quality of care (tips 10 - 12). Without due value of the paper’s figure, representing the professional attributes of both bars of the “T shaped healthcare professional”, a diagram might facilitate the organisation overview they propose as above and help the reader to keep these guidelines grouping in mind. Authors framed the engagement in IPE at the UGE level within the self-determination theory and provide an excellent explanation illustrated by daily practice examples. The patient’s perspective comes well integrated in IPE and is well highlighted throughout the paper. Authors justify a triangulation of fostering an autonomy supportive environment, the self-determination theory, and the development of competences of the T professional. IPE emerges as a methodology enabling the healthcare professionals to develop the competences needed to “collaborate inter-professionally with experts in other areas”, depicted the horizontal bar of the T shape. In Tip 11 we can find a delicious comparation of the concept of a puzzle with the sense of interdependent, complementary and collaborative work of the various healthcare professionals for the patients’ best outcome.The paper is robustly framed into learning theories and is judiciously combined with the Authors’ clinical and scholar experience in the field. Still, Authors are authentic and provide the reader with the complexity coupled with the advantages of an effective IPE. Their proposed guidelines will certainly inspire others to overcome challenges and barriers into developing a consistent and meaningful IPE project, starting in UGE.

Reviewer Expertise:

NA

No decision status is available

# Reviewer report: Reviewer response for version 1

**DOI:** 10.21956/mep.20183.r31212

**Published:** 2020-11-06

**Authors:** P Ravi Shankar

**Affiliations:** 1 American International Medical University

This review has been migrated. The reviewer awarded 4 stars out of 5 This is an interesting article on interprofessional education (IPE). IPE is not that common in Nepal, India or offshore Caribbean medical schools. Self-determination theory has an important role in enhancing motivation. IPE should strengthen autonomy and competence among different categories of students. The idea that IPE may reduce competence could be a reason why students may be reluctant to participate. The suggestion to Enhance collaboration among supervisors is an important point. If supervisors collaborate and understand well the benefits and challenges in doing so, they will be able to support IPE better. Ownership of learning can increase readiness for IPE and other learning situations. The T-shaped professional is a new but interesting concept for me. Balancing intraprofessional needs with interprofessional collaboration is very important. The paper will be useful to initiate and support IPE in different institutions.

Reviewer Expertise:

NA

No decision status is available
